# Understanding the role of total knee arthroplasty for primary treatment of tibial plateau fracture: a systematic review of the literature

**DOI:** 10.1186/s10195-020-00546-8

**Published:** 2020-05-25

**Authors:** Murray T. Wong, Jonathan Bourget-Murray, Kelly Johnston, Nicholas M. Desy

**Affiliations:** Section of Orthopaedic Surgery, Department of Surgery, University of Calgary, North Tower, Foothills Medical Centre, 1403 29 St NW, Calgary, T2N 2T9 AB Canada

**Keywords:** Total knee arthroplasty, Total knee replacement, Tibial plateau fracture, Complications, Outcomes

## Abstract

**Background:**

Surgical fixation of tibial plateau fracture in elderly patients with open reduction and internal fixation (ORIF) provides inferior outcomes compared with younger patients. Primary total knee arthroplasty (TKA) may be of benefit in elderly patients with a combination of osteoporotic bone and metaphyseal comminution. However, there continues to be conflicting evidence on the use of TKA for primary treatment of tibial plateau fracture. This systematic review was performed to quantify the outcomes and perioperative complication rates of TKA for primary treatment of tibial plateau fracture.

**Materials and methods:**

A comprehensive search of MEDLINE, Embase, and PubMed databases from inception through March 2018 was performed in accordance with PRISMA guidelines. Two reviewers independently screened papers for inclusion and identified studies featuring perioperative complications and outcomes of primary TKA for tibial plateau fracture. Weighted means and standard deviations are presented for each outcome.

**Results:**

Seven articles (105 patients) were eligible for inclusion. All-cause mortality was 4.75 ± 4.85%. The total complication rate was 15.2 ± 17.3%. Regarding outcomes, Knee Society scores were most commonly reported. The average Knee Society Knee Score was 85.6 ± 5.5, while the average Knee Society Function Score was 64.6 ± 13.7. Average range of motion at final follow-up was 107.5 ± 10.0°.

**Conclusions:**

Primary TKA for select tibial plateau fractures has acceptable clinical outcomes but does not appear to be superior to ORIF. It may be appropriate to treat certain geriatric patients with TKA to allow for early mobilization and reduce the need for reoperation. Other factors may need to be considered in deciding the optimal treatment.

**Level of evidence:**

Level III.

## Introduction

Fracture of the tibial plateau represents approximately 8% of fractures in elderly patients [1]. These injuries occur with an estimated incidence of 13.3 per 100,000 adults annually, with nearly a quarter of these occurring in elderly patients with concomitant osteoporosis [2, 3]. In young patients, the mainstay treatment for displaced tibial plateau fracture is open reduction and internal fixation (ORIF). However, in elderly patients with periarticular fracture, the combination of poor bone quality, metaphyseal bone comminution, and friable soft tissue envelope creates unique challenges to traditional ORIF. In these patients, surgical indications are controversial due to the increased risk of fixation failure. Published outcomes of operatively treated tibial plateau fracture in elderly patients have been largely inconsistent [4–6].

Interest, therefore, has developed in treating tibial plateau fracture in elderly patients with primary total knee arthroplasty (TKA). This approach offers the advantage of early full weight bearing and avoids some of the potential complications seen following ORIF. Additional benefits to treating select patients with TKA compared with ORIF are the reduced rates of lower-extremity thrombosis, postoperative pneumonia, and postoperative deconditioning [7, 8]. This is largely due to the immediate stability of the knee and early mobilization made possible following TKA. In addition, these patients require shorter hospital stays and fewer revision surgeries [9–11]. Although primary TKA may be a viable treatment option for comminuted intraarticular tibial plateau fracture in older patients, there continues to be controversy surrounding the use of TKA for primary treatment of tibial plateau fracture. Primary TKA for such fractures require revision-type implants, including stems, metaphyseal cones, or sleeves, and possibly increased levels of constraint and are, therefore, not straightforward primary knee arthroplasties.

Best practice recommendations remain unclear, and evidence-based guidelines are lacking. In deciding the optimal primary treatment for tibial plateau fracture, it is important to clarify the postoperative functional outcomes and complication rates following TKA to assess whether they are comparable to those of ORIF. This systematic review was performed to quantify the perioperative complication rates and clinical outcomes in patients following TKA for primary treatment of tibial plateau fracture. The objective is to establish whether TKA can be considered a reliable primary treatment option in select individuals.

## Materials and methods

### Search strategy and criteria

The present systematic review was performed in accordance with the Preferred Reporting Items for Systematic Reviews and Meta-Analyses (PRISMA) guidelines [12].

A comprehensive search for all level I–IV evidence published using the online databases MEDLINE, Embase, and PubMed was performed. The purpose of this search was to identify all eligible studies featuring perioperative complications or clinical outcomes in patients following TKA for primary treatment of tibial plateau fracture. The search terms and MeSH terms used were: (fracture, tibial[MeSH Terms]) OR (tibia* adj2 plateau adj3 fracture*) OR proximal tibia* fracture* AND (arthroplasty, knee replacement[MeSH Terms]) OR (total knee adj2 (replacement or arthroplast* or prosthe*)) OR TKA OR TKR AND (primary). All relevant articles published up to and including March 2018 were identified. Studies were included for final data analysis if they met the following criteria: (1) studies investigating TKA as initial treatment for tibial plateau fracture, (2) patients ≥ 55 years old, (3) minimum mean follow-up of 24 months, and (4) published in the English language. All prospective or retrospective studies, nonrandomized comparison studies, and case series were considered for inclusion. Articles were excluded if TKAs were performed in the context of revision surgery (prior ORIF or arthroplasty) or if fractures were determined to be pathologic. If more than one study was conducted at the same institution with duplicate subject publication, the article that had the most complete data was selected. Conference abstracts, gray literature, expert opinions, and review articles were excluded for the purpose of this study.

### Study selection

**Fig. 1:**
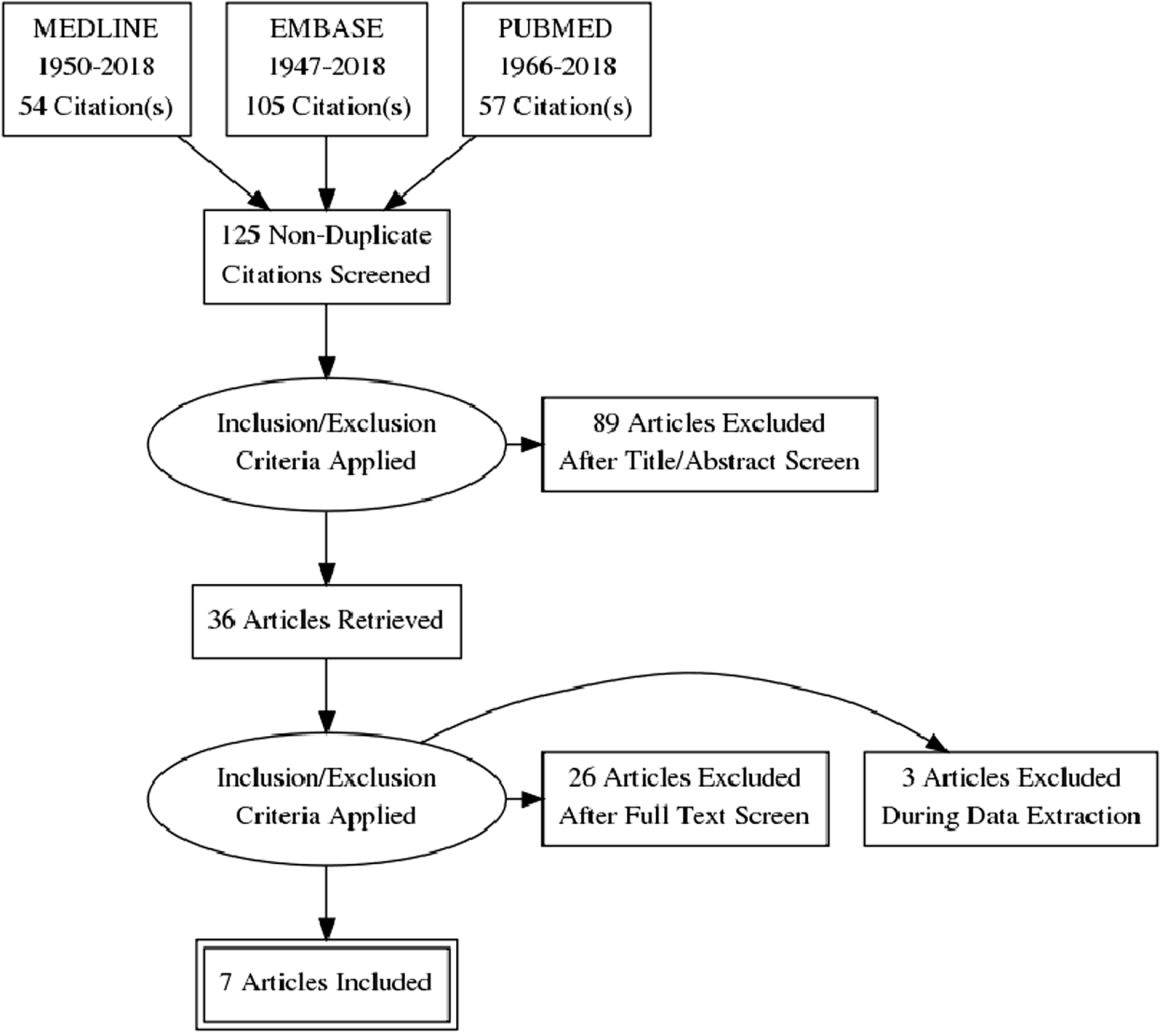
Flowchart showing search strategy and screening process. Seven studies were systematically reviewed for the purpose of this study

The search strategy yielded 216 potential articles, as outlined in Fig. 1. Duplicates were identified and removed using our reference manager, EndNote (Thomas Reuters, New York, NY). One hundred and five studies remained. Titles and articles were screened by two independent reviewers (MW and JBM) to determine study eligibility. The initial screen resulted in 36 articles that were subsequently retrieved (i.e., full-text manuscripts), independently reviewed, and accepted into the study if they met the inclusion criteria outlined above. In case of disagreement, the senior author (NMD) resolved the disagreement. Of the 36 full-text manuscripts reviewed, 7 studies were included in the final analysis. The reference lists of all seven studies were cross-referenced for articles that may not have been identified in the original search; no other studies were identified.

### Data extraction

Data extraction was performed by populating a predefined data abstraction sheet. Abstracted data included demographic information, enrollment period, mean age, male-to-female ratio, and average follow-up (Table 1). Outcomes of interest were mortality rates, perioperative complication rates, functional outcome, and postoperative knee flexion. All included studies are presented in Table 1.

**Table 1:** Patient demographics

Study	Year	Date range	No. patients	Age (years)	M:F	Follow-up in months (range)
Sarzaeem et al.	2017	2005–2009	30	68	19:11	54 (36–72)
Sivasubramanian et al.	2016	2010–2015	3	70	1:2	56 (18–82)
Huang et al.	2016	2010–2012	6	70	3:3	32 (25–41)
Haufe et al.	2016	2008–2014	30	78	13:17	27 (12–48)
Kini and Sathappan	2013	2007–2011	9	73	2:7	26 (14–38)
Malviya et al.	2011	2000–2008	15	77	2:13	47 (12–104)
Vermeire and Scheerlinck	2010	2002–2009	12	73	3:9	31 (5–81)
Total			105	72.7	43:62	39

### Statistical analysis

The summary statistics indicating the number of patients extracted from the individual studies were obtained using counts, frequencies, and percentages where necessary. Weighted means and standard deviations were calculated for each outcome. Metaanalysis could not be performed due to the heterogeneity in data across studies, and as a result, no statistical tests were performed.

## Results

### Demographics

Seven studies meeting the inclusion criteria were identified and included in our final analysis [10, 11, 13–17]. Collectively, these studies account for 105 patients. Among these studies, there were six retrospective case series (level IV) [10, 11, 13–16] and one prospective series [17]. No studies in this review compared primary TKA versus ORIF. The mean age of the patient population was 73 (range 58–93) years, and the average follow-up was 39 months (Table 1). Indications for primary TKA included advanced age, severe preexisting end-stage osteoarthritis (OA), poor bone quality or bone stock, or complex fractures in which ORIF was deemed too difficult. Based on the AO classification system, there was one 41A-type fracture, sixty-four 41B fractures, and thirty 41C fractures. Fifty-six patients (53%) were treated with posterior-stabilized or cruciate-retaining style implants, while 18 patients (17%) received constrained condylar implants and 31 patients (30%) received hinged implants. Of the 75 patients who had information available regarding stem implantation, 69 received a stemmed tibial component.

### Mortality

All seven studies reported mortality. The all-cause mortality rate over the follow-up period was 4.75 ± 4.85% (Table 2). Of the five deaths reported, two were described as unrelated to the operation, one occurred following a subsequent hip fracture, and two were associated with later operations on the same knee for prosthetic joint infection and periprosthetic fracture, respectively.

**Table 2:** Mortality, complication rates, functional outcome, and range of movement

Authors	All-cause mortality (%)	Complication rate	KSKS	KSFS	Postoperative knee flexion
Sarzaeem et al.	0	0	78	70	106°
Sivasubramanian et al.	0	33	90	N/A	122°
Huang et al.	0	0	84	N/A	119°
Haufe et al.	10	23	81	75	N/A
Kini and Sathappan	0	11	84	N/A	114°
Malviya et al.	6.7	6.7	90	42	92°
Vermeire and Scheerlinck	8.3	50	78	58	116°
Total	4.8 ± 4.9	15 ± 17	86 ± 6	65 ± 14	108 ± 10°

*KSKS* Knee Society Knee Score, *KSFS* Knee Society Function Score, *N/A*, not applicable

### Complications

All of the studies reported on perioperative complications but were heterogeneous in their descriptions. The total complication rate was 15.2 ± 17.3% (Table 2). A total of eight patients required revision surgery: three due to nonhealing wound, two for periprosthetic fracture, one due to a prosthetic joint infection, one required revision surgery to remove residual intraarticular cement, and one for aseptic loosening. Other complications included three superficial infections successfully treated with antibiotics, three hematomas which recovered spontaneously, one patient developed a symptomatic deep vein thrombosis, and one patient developed mild genu valgum.

### Outcomes

The most commonly reported outcome score was the Knee Society Score (KSS). The KSS involves 100 points each for the Knee Society Knee Score (KSKS) and Knee Society Function Score (KSFS), where 100 represents the best score [18]. This is a widely used two-part scale (KSKS and KSFS) designed specifically to evaluate outcomes after knee arthroplasty and has previously been validated [19]. The components of the KSKS encompasses pain, stability, and range of motion, while the KSFS assesses the ability to walk and climb stairs free of walking aids. Six of the seven studies (89 patients) reported the KSKS, while four studies (77 patients) reported the KSFS portion of the KSS. The average KSKS was 85.6 ± 5.5, while the KSFS was 64.6 ± 13.7 (Table 2).

### Clinical outcomes

Postoperative knee flexion was reported in six studies (72 patients). Range of motion was recorded upon the patient’s last follow-up. The average knee flexion achieved was 107.5 ± 10.0°, with a range from 80° to 130° (Table 2).

## Discussion

Interest in primary TKA for acute treatment of tibial plateau fracture has resurfaced in recent years, particularly with respect to elderly patients with complex periarticular fractures in whom ORIF may be associated with technical difficulties and increased perioperative complications. In fact, Ali et al. [20] reported an overall 31% rate of failure of fixation, being 79% in patients older than 60 years compared with 7% in younger patients, and 100% in patients with significant osteoporosis. The parameters that were statistically significantly associated with loss of reduction were age above 60 years, premature weight bearing, preoperative displacement, fracture fragmentation, and severe osteoporosis. For these reasons, TKA offers the theoretical advantage of immediate stability, early full weight bearing, and decreased risk of requiring revision surgery [7]. However, controversy persists surrounding the role of TKA for primary treatment of tibial plateau fracture. This systematic review was performed to assess the functional outcomes and complications of TKA when used to definitively treat tibial plateau fracture.

The results of this systematic review show that, while acceptable results may be achieved with TKA for primary treatment of tibial plateau fracture, complication rates remain high (15.2 ± 17.3%). Meanwhile, the mean KSKS was 85.6 ± 5.5, which is deemed excellent as per the predefined grading scheme for the KSS (score 80–100, excellent; score 70–79, good; score 60–69, fair; score < 60, poor). In light of this, none of the included studies reported, on average, less than a good score [21]. The KSFS was reported by fewer articles but was 64.6 ± 13.7, indicating a fair result. With respect to the KSFS, two studies did report poor overall patient function (score < 60) [11, 13]. Sarzeem et al. [17], who carried out the only prospective series included in this systematic review, reported excellent results on the KSKS (90.7 ± 6.5) and fair results on the KSFS (69.6 ± 8.8). In this series, all 30 consecutive patients over 60 years of age returned to their previous activities, and none experienced any postoperative infection, thromboembolic events, or aseptic loosening.

From a functional standpoint, our data show a mean postoperative knee flexion of 108° following TKA, which is near 110°, a threshold used to consider the result of elective TKA to be good [22]. The poorest range of motion reported across all the studies, in one patient, was 80°. In contrast, a series of 43 patients who underwent ORIF for tibial plateau fracture were found to have a mean knee flexion of 128° following unicondylar tibial plateau fixation and 110° following bicondylar tibial plateau fixation [23]. Therefore, TKA may provide a range of motion similar to ORIF following fixation of more complex (bicondylar) tibial plateau fractures.

Complication rates following ORIF for tibial plateau fracture range from 10% to 15%, which is less than what we report following primary TKA. However, 25–45% of patients with tibial plateau fractures treated with ORIF eventually develop radiological evidence of posttraumatic arthritis upon long-term follow-up [24–26]. However, only 15% of these patients experience enough symptoms to necessitate revision surgery to a TKA [5, 9, 20, 24, 27–30]. Wasserstein et al. [27] identified 8426 patients (mean age 48.89 ± 16.88 years) who underwent ORIF for tibial plateau fracture and found a 5.3% rate of conversion to TKA at 5 years and 7.3% at 10 years, with a calculated hazard ratio of 1.034 for each year over the age of 48 years. Patients were also more likely to require TKA if they were female (hazard ratio 1.25), had a greater number of comorbidities (hazard ratio 2.17), or suffered bicondylar fracture (hazard ratio 1.53) [27]. Studies have found that 72–90% of patients who receive ORIF for tibial plateau fracture report good to excellent functional outcomes using the Rasmussen clinical scale [4–6, 26].

The outcomes of TKA for posttraumatic arthritis are less reliable than for idiopathic end-stage primary OA [25, 31, 32], with perioperative complication rates ranging from 14% to 67% [33]. Lunebourg et al. found that clinical outcomes and implant survival after TKA for posttraumatic arthritis were lower than after TKA done for primary OA at mean follow-up of 11 (5–15) years: the patients’ mean KSKS was 77 versus 87, while the mean KSFS was 81 versus 89 [34]. The survival rate of TKA at 10 years showed better results in the primary OA group (99% versus 79%; *p* < 0.001), with reoperation mainly performed within the first 2 years after surgery. These findings are in keeping with other studies: Lizaur-Utrilla et al. [35] reported a 13.7% complication rate when TKA was performed in the context of posttraumatic arthritis (initial treatment: ORIF, 22 knees; nonoperative management, 7 knees) compared with 0% for patients who underwent TKA for end-stage primary OA. Similarly, Scott et al. [28] reported that complication rates were higher in the posttraumatic arthritis cohort than when undertaken for primary OA (of the 31 patients: ORIF, 24 patients; treated nonoperatively, 7 patients), and included wound complications (13% versus 1% *p* = 0.014) and persistent stiffness (10% versus 0%, *p* = 0.014). However, it is critical to understand the difference between patients who are initially treated with ORIF versus managed nonsurgically and go on to develop posttraumatic arthritis needing TKA. In 2015, Abdel et al. [36] reported the 15-year outcomes after TKA in 62 patients with prior tibial plateau fracture. Comparing patients who were originally treated with ORIF (38 patients) with those managed nonoperatively (23 patients), they found no significant difference in perioperative complications, postoperative pain, or postoperative KSS.

Performing TKA in the context of acute tibial plateau fracture or in patients with previous ORIF can require higher-constraint components and possibly stems and/or metaphyseal augments (e.g., cones and sleeves), making these surgeries technically more difficult and thus behaving more like revision TKA for failed arthroplasty. Several studies have reported the use of stemmed prosthesis for management of acute periarticular proximal tibial plateau fracture [11, 13, 32, 37, 38]. Some authors advocate the use of constrained or hinged prostheses for these complex fractures, as this facilitates any difficulty with soft-tissue balancing and bone loss [1, 13, 32, 38]. It is imperative to restore lower limb axial alignment, as implant malposition and residual deformity have previously been shown to result in poorer outcomes [25].

The immediate management of acute tibial plateau fracture in elderly patients continues to be controversial. Although there is emerging evidence showing positive results for primary TKA for tibial plateau fracture, it is paramount to appreciate that performing TKA in the context of acute tibial plateau fracture is not a straightforward endeavor. Many of these patients have notable loss of the articular surface, which disrupts normal anatomic references, and compromised soft tissue due to possible coexisting ligamentous injury [11]. It is the authors’ recommendation that any TKA in the context of acute tibial plateau fracture be performed by experienced knee arthroplasty surgeons.

While acceptable results may be achieved with TKA for acute tibial plateau fracture, the complication rate is unacceptably high. Further prospective, preferably randomized clinical trials, and long-term follow-up data will be necessary to prove the ultimate benefits of this treatment strategy. It is the authors’ opinion that initial conservative management or ORIF can be helpful to restore axial alignment and bone stock, and few patients will ultimately require conversion to TKA.

The limitations of this review include the retrospective nature of the studies included, along with lack of control groups across the studies. In addition, varied reporting of complication rates and heterogeneity of results led to imprecise estimates of true outcomes. These outcomes are difficult to compare with those of patients treated with ORIF or delayed arthroplasty as the literature reports on younger populations and includes patients with tibial plateau fracture initially treated nonoperatively. Also, we were unable to report on quality of life or patient satisfaction with surgical outcomes, as the included studies did not consistently report this information. In addition, it is important to recognize that the only prospective study included in this systematic review contributed 29% of the patients. This may create a possible source of selection bias. This review presents the best available evidence with mean follow-up of 39 months, but longer-term outcomes after primary TKA for tibial plateau fracture must still be studied to fully understand implant survivorship in this clinical context. Strengths of the study, on the other hand, are the comprehensive review of current literature in an area of growing interest, and the estimates of mortality, complication rates, and functional outcomes, which can be used to guide treatment decisions and power future studies.

In conclusion, total knee arthroplasty for treatment of acute tibial plateau fracture is an enticing alternative to ORIF to allow early weight bearing, especially in elderly patients with poor bone quality and a friable soft tissue envelope. However, our systematic review shows that complication rates remain high, functional scores are only average, and knee range of motion is not any better than ORIF when TKA is performed for acute tibial plateau fracture. Given these findings, surgeons should consider nonoperative care or ORIF as first-line treatment for acute tibial plateau fracture in elderly patients. More studies are needed to further elucidate the potential use of TKA as initial treatment for certain acute tibial plateau fractures in select individuals.

## Data Availability

All data presented and available in primary articles.

## Abbreviations

ORIF: Open reduction and internal fixation
TKA: Total knee arthroplasty
PRISMA: Preferred Reporting Items for Systematic Reviews and Meta-Analyses
OA: Osteoarthritis
KSS: Knee Society Score
KSKS: Knee Society Knee Score
KSFS: Knee Society Function Score
